# Divergent accumulation of microbial necromass and plant lignin components in grassland soils

**DOI:** 10.1038/s41467-018-05891-1

**Published:** 2018-08-28

**Authors:** Tian Ma, Shanshan Zhu, Zhiheng Wang, Dima Chen, Guohua Dai, Bowei Feng, Xiangyan Su, Huifeng Hu, Kaihui Li, Wenxuan Han, Chao Liang, Yongfei Bai, Xiaojuan Feng

**Affiliations:** 1 State Key Laboratory of Vegetation and Environmental Change, Institute of Botany, Chinese Academy of Sciences, Beijing, 100093 China; 2 College of Resources and Environment, University of Chinese Academy of Sciences, Beijing, 100049 China; 3 Department of Ecology, College of Urban and Environmental Sciences, Peking University, Beijing, 100871 China; 4 Xinjiang Institute of Ecology and Geography, Chinese Academy of Sciences, Urumqi, 830011 China; 5 College of Resources and Environmental Sciences, China Agricultural University, Beijing, 100193 China; 6 Institue of Applied Ecology, Chinese Academy of Sciences, Shenyang, 110016 China

## Abstract

The means through which microbes and plants contribute to soil organic carbon (SOC) accumulation remain elusive due to challenges in disentangling the complex components of SOC. Here we use amino sugars and lignin phenols as tracers for microbial necromass and plant lignin components, respectively, and investigate their distribution in the surface soils across Mongolian grasslands in comparison with published data for other grassland soils of the world. While lignin phenols decrease, amino sugars increase with SOC contents in all examined grassland soils, providing continental-scale evidence for the key role of microbial necromass in SOC accumulation. Moreover, in contrast to clay’s control on amino sugar accumulation in fine-textured soils, aridity plays a central role in amino sugar accrual and lignin decomposition in the coarse-textured Mongolian soils. Hence, aridity shifts may have differential impacts on microbial-mediated SOC accumulation in grassland soils of varied textures.

## Abstract (web summary)

It remains unclear how microbes and plants contribute to soil organic carbon (SOC) accrual. Here, using biomarkers, the authors show that microbial necromass and plant-derived lignin components have divergent accumulation mechanisms and that microbial necromass plays a key role in SOC accumulation.

## Introduction

Organic carbon with slow (i.e., centennial to millennial) turnover times constitutes the largest fraction of soil carbon^1,2^. How microbes and plants contribute to the formation and accumulation of these soil organic carbon (SOC) pools is a fundamental and much-debated question related to soil carbon dynamics and response to global changes^1–4^. Conventionally, plant structural compounds such as lignin are considered to be a key contributor to the slowly cycling SOC due to their chemical recalcitrance^1,5^ and accumulation in decaying litter^6^. However, mounting evidence shows that lignin prevails mainly in particulates as plant debris rather than preserved in mineral soils^7,8^ and is a relatively small component in the mineral-associated and old soil fractions^9,10^, indicating that lignin may not be as important in SOC accumulation as previously thought.

The emergent consensus is that microbial-derived carbon plays a more important role in the buildup of slowly cycling SOC^3,4,11–13^. With the decay of plant materials, soil microbes convert available carbon into microbial necromass or microbial-processed compounds along with their own biomass. As microorganisms tend to attach to surfaces, microbial residues accumulate on mineral-associated soil fractions^9,11^. While microbial biomass has a fast turnover and constitutes a tiny fraction of SOC, microbial necromass is considered to be relatively stable^14^ and accrue in the soil with iterative community turnover^4,11,13,15,16^. This process, embedded in the microbial carbon pump originally put forward by marine researchers^17^, is considered to be a key mechanism contributing to the persistence of organic carbon in soils as well^18,19^. However, this mechanism has not been validated on landscape scales so far due to limited analytical tools to separate microbial-derived vs. plant-derived carbon in natural soils. Even less is known about environmental controls on the preservation of microbial-derived and plant-derived components or the optimal conditions for microbial sequestration of SOC. Filling these knowledge gaps will not only help to decipher the fate of SOC under global changes but also provide conceptual guidance for the development of soil carbon models incorporating microbial processes.

Here we utilize two groups of widely accepted biomarkers (i.e., amino sugars and lignin phenols) to trace microbial necromass^14,20,21^ and plant-derived lignin components^7,22^, respectively. We compare their distribution in the surface soils (0–10 cm) of 38 replicated sites across two large-scale transects of Mongolian grasslands (involving a total of 113 amino sugars and 39 lignin phenol samples), coupled with lignin analysis for the typical vegetation overlaying different grasslands. To complement this dataset based on poorly-weathered and coarse-textured Mongolian soils^23^, we further compile all the published data of amino sugars and lignin phenols in grassland surface soils (0–10 cm) elsewhere using similar analytical methods (including 54 and 70 concentration data for amino sugars and lignin phenols, respectively). Using statistical analysis encompassing similar environmental variables, we compare mechanisms regulating the accumulation of both biomarkers in the Mongolian grasslands and contrast environmental influences on amino sugar accrual in the coarse-textured Mongolian soils vs. fine-textured grassland soils elsewhere. This approach allows us to assess the large-scale distribution and preservation of microbial necromass vs. plant lignin components in grassland soils spanning an unprecedented range of environmental gradients. Overall, we demonstrate coupled variations of amino sugars rather than lignin phenols with SOC in all examined grassland soils and highlight the critical role of aridity in amino sugar accrual and lignin decomposition in the coarse-textured Mongolian soils in contrast to fine-textured soils. Our results provide proof-of-concept evidence for the key role of microbial necromass in SOC accumulation and suggest that aridity shifts may have differential impacts on microbial-mediated SOC accumulation in grassland soils of varied textures.

## Results

### Distribution of amino sugars and lignin phenols

**Fig. 1:**
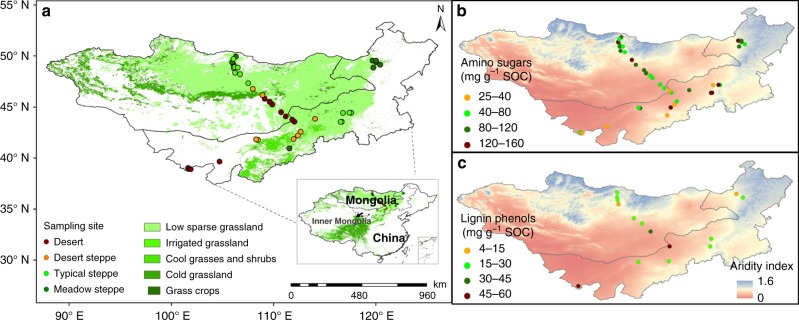
Sampling sites and biomarker concentrations. Spatial distribution of sampling sites across the Mongolian grasslands (**a**) and soil organic carbon (SOC)-normalized concentrations of amino sugars (**b**) and lignin phenols (**c**) in the surface soils. Land cover classification is based on the Global Land Cover Characteristics Database v2.0 (https://lta.cr.usgs.gov/GLCC). Data for aridity index are obtained from Global Aridity and PET Database (http://www.cgiar-csi.org/data/global-aridity-and-pet-database)

**Fig. 2:**
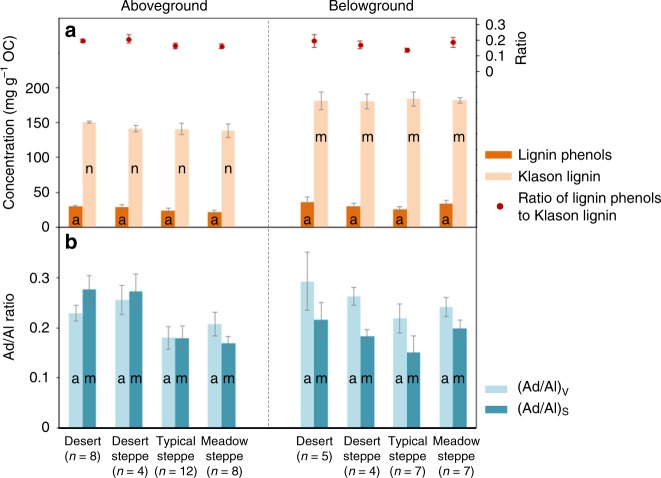
Lignin concentration and composition in the overlaying vegetation of Mongolian grasslands. Organic carbon (OC)-normalized concentrations of lignin phenols and Klason lignin and the ratio of lignin phenols: Klason lignin (**a**) and the acid-to-aldehyde (Ad/Al) ratios of vanillyl (V) and syringyl (S) phenols (**b**). Error bars represent the standard error of the mean (s.e.m.) with the number of replicates (n) indicated in the parenthesis. Letters indicate different levels for the same parameter among vegetation types (*p* *<* 0.05)

**Fig. 3:**
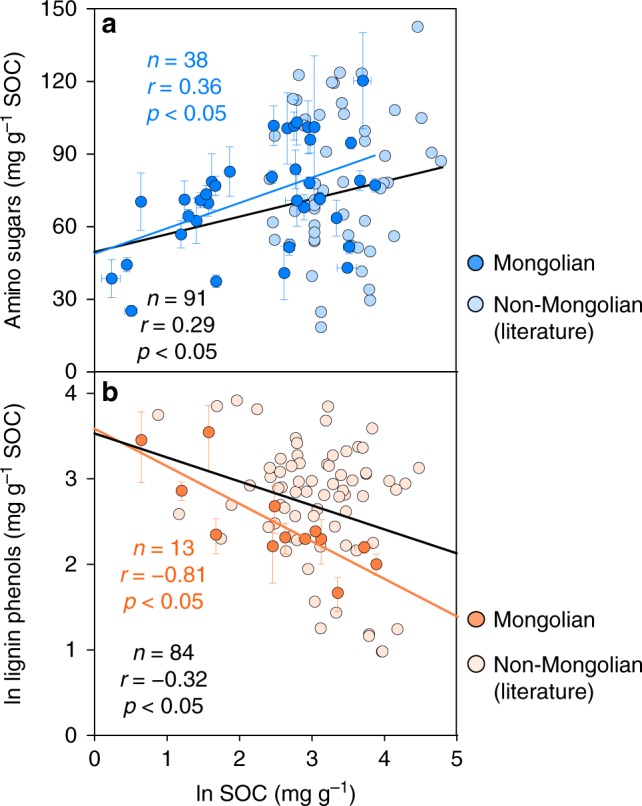
Relationships between biomarker concentrations and soil organic carbon (SOC) contents. Pearson correlations between SOC-normalized concentrations of amino sugars (**a**) and lignin phenols (**b**) with SOC content in the surface soils of Mongolian (dark colored dots) and non-Mongolian grasslands (from the literature in Supplementary Data 2; light colored dots). Colored and black lines indicate linear regressions for the Mongolian and all combined data, respectively. Error bars represent s.e.m. for three site replicates of the Mongolian soils

In the surface soil of Mongolian grasslands (Fig. 1a), amino sugars had an SOC-normalized concentration of 21–158 mg g^−1^ SOC (Fig. 1b; Supplementary Data 1), dominated by glucosamine. This is consistent with the literature data exhibiting a similar range of concentration in the surface soil of grasslands elsewhere^14,20,24,25^ (18–155 mg g^−1^ SOC; full list of references in Supplementary Data 2; Supplementary Fig. 1). By comparison, lignin phenols represented a smaller proportion of SOC (4–60 mg g^−1^ SOC; Fig. 1c), also in line with the literature data^26–29^ (full list of references in Supplementary Data 2). Notably, amino sugars and lignin phenols displayed contrasting distribution patterns across the Mongolian transects (Fig. 1b, c). Compared with sites under other vegetation types, soils in the arid deserts had a higher concentration of lignin phenols and a lower abundance of amino sugars per unit of SOC (*p* < 0.05). This difference is not related to the chemical composition of vegetation covering varied sites, since neither ratios of organic carbon-to-nitrogen (N; Supplementary Fig. 2) nor lignin contents (assessed by both lignin phenols and Klason lignin;^30^ Fig. 2a) per unit of organic carbon differed in the overlaying plants of different vegetation types (*p* > 0.05; Supplementary Data 3). The ratio of lignin phenols to Klason lignin, ranging from 0.06 to 0.30, does not differ among vegetation types, either (*p* > 0.05; Fig. 2a). Hence, yields of lignin phenols from the lignin macromolecule are comparable across our transects. Furthermore, although lignin phenols and amino sugars do not show significant correlations (*p* > 0.05) possibly due to variation of other SOC components (such as black carbon that is prevalent in grassland soil^31^), the SOC-normalized concentrations for these two groups of biomarkers show an opposite correlation with SOC contents in the surface soil of Mongolian grasslands, as well as all grasslands compiled (Fig. 3): while amino sugars increase with SOC concentrations (*n* = 91; *p* < 0.05), lignin phenols decrease (*n* = 84; *p* < 0.05).

**Fig. 4:**
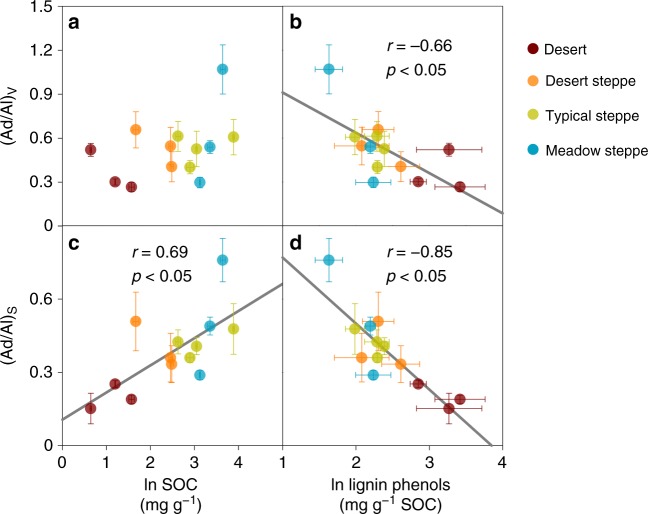
Variations of lignin acid-to-aldehyde (Ad/Al) ratios in the Mongolian grassland soils. Pearson correlations of the Ad/Al ratios for vanillyl (V) and syringyl (S) phenols with soil organic carbon (SOC; **a**, **c**) and lignin phenol concentrations (**b**, **d**). Error bars represent s.e.m. for three site replicates. Black lines indicate linear regressions (*p* < 0.05; *n* = 13)

Lignin degradation is further assessed by the acid-to-aldehyde (Ad/Al) ratios of vanillyl (V) and syringyl (S) phenols, which typically increase with increasing lignin oxidation^22,32^. The aboveground biomass of the overlaying plants showed lower (Ad/Al)_S_ but similar (Ad/Al)_V_ values in the meadow steppes relative to other vegetation types along the Mongolian transect (Fig. 2b), while the belowground biomass had similar Ad/Al ratios among all vegetation types (*p* > 0.05). In contrast, desert (rather than meadow steppe) soils had a lower (Ad/Al)_S_ ratio relative to all other soils (*p* < 0.05). Moreover, both Ad/Al ratios increase with increasing SOC content and with decreasing lignin phenol concentrations in the Mongolian soils (*p* < 0.05; Fig. 4), confirming a lower lignin oxidation stage in soils with a lower SOC content and a higher lignin phenol concentration.

### Environmental influences in Mongolian soils

To further explore environmental influences on the distribution of amino sugars vs. lignin phenols in soils, a detailed list of 12 variables were measured or compiled for the Mongolian grasslands, including regional aridity (assessed by aridity index as the ratio of annual precipitation to potential evapotranspiration^33^), plant aboveground and belowground biomass, microbial biomass (represented by phospholipid fatty acids; PLFAs^34^), soil texture (especially clay), SOC, N, soil pH, reactive iron (Fe), and aluminum (Al) contents, etc. Amino sugars and lignin phenols display opposite correlations with the majority of the investigated variables (Supplementary Figs. 3-4), corroborating their differential stabilization mechanisms in the soil.

To delineate the accumulation mechanisms, structural equation modeling (SEM) was employed to quantify the complex interactions between biomarker concentrations and environmental variables. The latter is grouped into six categories: climate (represented by aridity index), plant (including aboveground and belowground biomass), microbial biomass (represented by PLFAs), soil C and N (including SOC and N), pH and mineral (i.e., clay for amino sugars to compare with the literature data^35^ and reactive Fe and Al for lignin phenols^36^). The SEM is developed from a priori models (Supplementary Fig. 5) based on knowledge, with potential flows of causality from all categories of variables to the dependent biomarkers and from climate to all other variables except soil mineral (which strongly depends on parent materials^37^). The validated SEMs yield a good model fit, indicated by a non-significant *Χ*^2^ test (*P* > 0.05), a high comparative fit index (CFI > 0.95), a low root mean square error of approximation (RMSEA < 0.05)^38^.

**Fig. 5:**
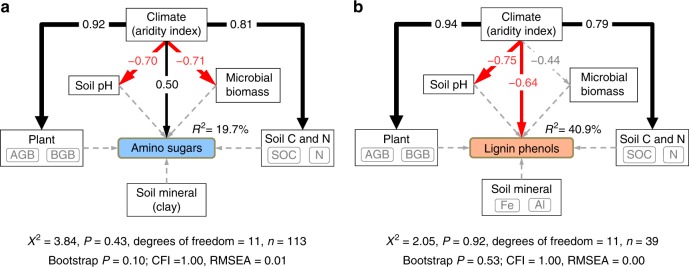
Cascading relationships of amino sugars and lignin phenols with environmental variables. Best-supported structural equation models disentangling major pathways of environmental influences on the soil organic carbon (SOC)-normalized concentrations of amino sugars (**a**) and lignin phenols (**b**) in the Mongolian grasslands with block effect accounted for. Black and red arrows indicate positive and negative flows of causality (*p* < 0.05), respectively. Gray dotted lines indicate insignificant pathways from a priori models (Supplementary Fig. 5). Numbers on the arrow indicate significant standardized path coefficients, proportional to the arrow width. R^2^ indicates the variance of biomarkers explained by the model. Environmental variables are categorized into plant, soil carbon (C) and nitrogen (N), and soil mineral by a principle component analysis (PCA). Variables in the gray boxes show a positive correlation with the corresponding primary environmental category (Supplementary Table 6). Microbial biomass is represented by phospholipid fatty acids. AGB aboveground biomass, BGB belowground biomass, Fe iron, Al aluminum

Based on the SEM, aridity index, rather than edaphic properties, exerts a direct and dominant effect on the concentration of both biomarkers across the Mongolian grasslands, explaining 19.7% and 40.9% of variation for amino sugars and lignin phenols, respectively, albeit in opposite directions (Fig. 5a, b). As amino sugars increase with increasing aridity index (indicating decreasing aridity), lignin phenols decrease (*p* < 0.05). The dominant influence of aridity is supported by multiple stepwise regression analyses (where aridity index is the only variable retained in the model; Supplementary Table 1) and partial correlations: none of the variables have any effect on the concentration of either biomarker after the effect of aridity index is accounted for (*p* > 0.05). In contrast, aridity index remains significantly correlated with both biomarkers after the effect of other variables (except SOC and N for amino sugars) is accounted for (*p* < 0.05; Supplementary Tables 2-3). Moreover, aridity’s effect on lignin phenol distribution in the soil is not related to lignin phenol abundances in the overlaying vegetation, which show no correlation with aridity index (*p* > 0.05; Supplementary Fig. 6a).

To further confirm aridity’s control on lignin degradation, we used multiple stepwise regression analysis to investigate environmental influences on the Ad/Al ratios in the Mongolian soils. The (Ad/Al)_V_ ratio, which is negatively (rather than positively) related to aridity index in plant biomass (*p* < 0.05; Supplementary Fig. 6b), does not correlate to any variables in the soil (*p* > 0.05; Supplementary Fig. 7). However, among all the variables showing significant correlations with the (Ad/Al)_S_ ratio (including aridity index, aboveground biomass, SOC and N contents; *p* *<* 0.05; Supplementary Fig. 7), aridity index is the single most important variable, showing a positive effect on the ratio (Supplementary Table 4). Again, this effect is not related to lignin composition of the overlaying vegetation, as the (Ad/Al)_S_ ratio of plant biomass shows no correlation with aridity index (*p* > 0.05; Supplementary Fig. 6c).

In addition, total and individual amino sugars (except muramic acid that exclusively derived from bacteria^39,40^) are negatively related with total and subgroups (i.e., fungal, gram-positive, and gram-negative bacterial) of PLFAs across the Mongolian grasslands by correlation analysis (*p* < 0.05; Supplementary Fig. 8) but they are not connected in the SEM (Fig. 5a). These results suggest that microbial necromass distribution varies from that of living microbial community as microbial biomass may be more prone to seasonal variations compared with its necromass. Similarly, lignin phenol concentration in the surface soils are not related to plant biomass distribution (Fig. 5b), suggesting that lignin preservation rather than input determines its abundance in the Mongolian soils.

### Divergent controls on amino sugar accrual

**Fig. 6:**
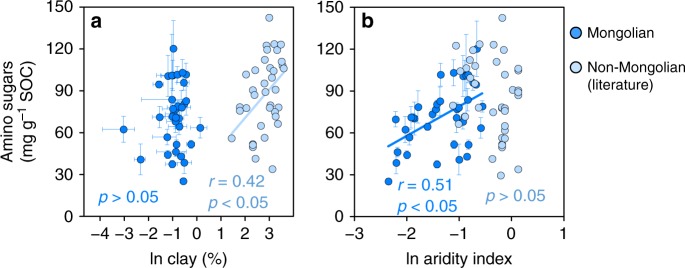
Variations of amino sugar concentrations in different soils. Pearson correlation with clay contents (**a**) and aridity index (**b**) in the Mongolian (*n* = 38) and non-Mongolian grassland soils (*n* = 38). SOC, soil organic carbon. Error bars represent s.e.m. for three site replicates in the Mongolian grasslands. Data for non-Mongolian soils are derived from the literature^14,20,24,25^, which uses a different clay measurement method from the Mongolian soils

In contrast to the Mongolian soils, amino sugars in the surface soils of non-Mongolian grasslands are only and positively correlated with clay contents (*p* < 0.05; Fig. 6a) among the examined variables (including soil pH, SOC, and N contents; Supplementary Data 2) and not influenced by aridity index (*p* > 0.05; Fig. 6b). It is notable that the Mongolian soils exhibit much lower clay contents (0.44 ± 0.03%; *n* = 38) relative to those in the literature (i.e., grasslands in the US Great Plains^14,20,24^ and Germany^25^) (18.5 ± 1.42%; *n* = 38). Admittedly, the laser diffraction method that we employed, although yielding similar clay contents for the Mongolian soils as in other studies^41,42^, tends to underestimate fine particles compared to the sieving-centrifugation method used by the non-Mongolian studies^43,44^. However, using the same hydrometer-based method, Evans et al.^45^ have confirmed that soils in the Mongolian grasslands are coarser (with an average clay content of 12.5%) than those in the US Great Plains (average clay content of 27%). Hence, it appears that aridity’s influence is dampened by clay’s protective effect on amino sugars in the fine-textured soils.

## Discussion

This study represents the first attempt to compare the distribution of microbial necromass vs. plant lignin components in grassland soils over regional scales. The contrasting distribution pattern of amino sugars vs. lignin phenols in relation to SOC contents (Fig. 3) implies an enrichment of microbial necromass at the expense of plant lignin components with SOC accrual^3,4,11,13,15^. The decrease of lignin phenols is accompanied by an increase of Ad/Al ratios across the grassland transects (Fig. 4) despite relatively invariant lignin composition in the overlaying vegetation (Fig. 2), corroborating enhanced lignin oxidation in SOC-rich soils. Although not reported previously, the correlations of lignin Ad/Al ratios with SOC contents^26^ and lignin phenol concentrations^26–28^ were present in some but not all grassland studies^29^ (*p* < 0.05; Supplementary Fig. 9). Variations in lignin composition of vegetation and/or decomposition processes among different ecosystems may contribute to the divergent relationships. In contrast to lignin phenols, the positive correlation between amino sugars and SOC contents in all examined grassland soils (Fig. 3a) provides unequivocal evidence for the coupled accumulation of microbial necromass (instead of lignin) with SOC at a broader scale. This striking contrast has not been reported previously^5,20^ partly due to the limited range of environmental variables studied in small-scale investigations.

Furthermore, using SEM encompassing a comprehensive list of environmental variables, we show that amino sugar accumulation and lignin decomposition are mainly controlled by climate (i.e., aridity) in the coarse-textured Mongolian grassland soils (Fig. 5a, b). Aridity’s control on lignin decay is further supported by the dominant influence of aridity index on the (Ad/Al)_S_ ratio (Supplementary Table 4). Given that fungal PLFAs increase relative to bacterial PLFAs with increasing aridity and pH across the Mongolian transects^34^, lignin decay is not enhanced by the dominance of fungi (as the primary decomposer of lignin^46^) in the microbial communities in drier soils. Instead, our results imply that in the semi-arid Mongolian grassland soils, lignin decomposition increases with increasing moisture, which promotes microbial activity (and amino sugar accumulation). This conclusion is reasonable given that moisture is the primary limiting factor for decomposition processes in arid and semiarid regions^47^. Microbial growth and activity are both known to decrease under arid conditions^48^, thereby lessening plant carbon decay and its conversion into microbial carbon. Moreover, microbial necromass was reported to decrease under drought^49^, further contributing to the decline of amino sugars with increasing aridity. It is also notable that the distribution of total amino sugars is different from that of PLFAs (Supplementary Fig. 8). As living microbial biomass is relatively minor compared with microbial necromass in the soil^14^, microbial turnover^12,50^ and/or necromass preservation efficiency^12^ may play a more important role in amino sugar accrual in the soil. This finding agrees with the notion that amino sugars inform on soil legacy while PLFAs offer a snapshot of current microbial communities^14,39,51^.

In contrast, clay content exerts a key influence on amino sugar abundance in non-Mongolian grassland soils with a relatively higher content of clay (Fig. 6). This finding is consistent with the conclusion of Six et al.^35^, presumably due to physical protection of necromass against microbial re-utilization^52^ provided by sorption to fine-sized particles^53^. This effect is not observed in the Mongolian soils mainly due to their coarse textures, thereby lacking physical and chemical protection mechanisms for the accumulation of microbial-derived carbon via sorption and/or aggregation^53–55^. Hence, our results indicate divergent controls on amino sugar accumulation in grassland soils of varied textures. While mineral-organic interactions play an important role in the stabilization of microbial necromass in fine-textured soils, microbial-mediated carbon accumulation is regulated by climate (i.e., aridity) in the coarse-textured soils of drylands. Hence, aridity shifts may have differential impacts on microbial sequestration of SOC in grasslands with different soil textures.

In summary, our study provides proof-of-concept evidence for the contribution of microbial necromass to SOC accumulation in grasslands over regional scales and highlights the role of aridity in amino sugar accrual and lignin decomposition in the coarse-textured Mongolian soils covered by vegetation with relatively uniform lignin concentrations and compositions (Fig. 2). It should be emphasized that aridity’s control on microbial necromass accumulation has its operating ranges such that its effect is dwarfed by clay’s influence on amino sugars in the fine-textured soils of non-Mongolian grasslands (Fig. 6a) through protection by sorption and/or aggregation^54,55^. In addition, it should be mentioned that other variables may also play an important role in microbial carbon stabilization under different environments, including nutrient availability and substrate quality^11,56^, etc. This is partially reflected by the relatively low amount of variation explained by the SEM for amino sugars (Fig. 5a). For instance, in humid and sub-humid forest ecosystems where moisture does not limit microbial growth and activity, N availability and lignin contents of the decaying litter may affect microbial carbon use efficiency^57^ and/or re-mineralization of microbial necromass^58^, thereby exerting a stronger influence on microbial carbon accrual in the soil. Hence, further efforts are needed to investigate and compare factors controlling amino sugar accumulation in different soils and ecosystems to better elucidate microbial sequestration of soil carbon.

## Methods

### Soil sampling in the Mongolian grasslands

Two grassland-dominated transects in the central part of the Eurasian steppe are included in this study^34^, spanning ~2000 km from east to west and ~900 km from north to south (Fig. 1a). Mean annual precipitation ranges from 104 to 412 mm while mean annual temperature ranges from –2.8 to 8.1 °C (obtained from the WorldClim-Global Climate Data; http://www.worldclim.org), resulting in a sharp aridity gradient across the transects (Supplementary Data 1). Aridity index is used as an integrated index of climatic aridity and obtained from the Global Aridity and PET Database (http://www.cgiar-csi.org/data/global-aridity-and-pet-database). Each transect encompasses four major vegetation and soil types^59^, namely meadow steppe (Calcic Chernozem), typical steppe (Kastanozems), desert steppe (Calcisols), and desert (Calcisols). Soils are poorly weathered with a low value (~53) for the chemical index of alteration^23^.

In the summer of 2010 and 2011, surface soils (0–10 cm) were collected from 38 fully replicated sites along the transects using soil cores (diameter of 7 mm). At each site, five quadrats (1 × 1 m) were randomly selected within a 100 × 100 m area with distance of >20 m between each other. Three soil cores were taken from the 0–20 cm depth in each quadrat and split into two depths (0–10 and 10–20 cm). Soils from the same depth were mixed in situ for each quadrat as one composite sample and each quadrat served as a replicate for analysis, given the large heterogeneity of SOC composition and microenvironments experienced by soil microbes. We randomly selected three out of five replicates at each site for amino sugar analysis (except one site where only two replicates were available) while 13 sites (with three replicates each) were chosen for lignin phenol analysis due to limited sample availability. As a result, a total of 113 and 39 surface soil samples were selected for amino sugar and lignin phenol analyses (Supplementary Data 1). Aboveground and belowground biomass was determined at each quadrat according to Chen et al.^34^. Concentrations of PLFAs were measured for freshly freeze-dried samples by Chen et al.^59^.

As plant samples from the original sampling campaign were not kept, we also collected biomass of the overlaying vegetation from various sites under four vegetation types on the Mongolian Plateau in the summer of 2017. These samples included 32 aboveground and 23 belowground (0–10 cm) biomass samples, all of mixed species collected using similar protocols as in Chen et al.^34^. (Supplementary Data 3). Biomass samples were kept at 4 °C before returning to the laboratory. After cleaning, all plant tissues were oven-dried at 65 °C and crushed with a ball mill prior to chemical analysis.

### Soil bulk properties

Soils were homogenized, air-dried immediately after sampling and passed through a 2-mm sieve with roots removed before analysis. Soil pH was measured at a soil:water ratio of 1:2.5 (w:v). SOC and total N contents were determined using the Walkley–Black modified acid-dichromate ferrous sulfate titration method and micro-Kjeldahl digestion method coupled with colorimetric determination^60^. Organic C and N contents of plant samples were measured by combustion using an elemental analyzer (Vario EL III, Elementar, Hanau, Germany). Reactive Fe, Al were extracted by the citrate-bicarbonate-dithionite method^61^ and measured on the inductive coupled plasma emission spectrometer (Thermo ICAP6300, USA). To measure soil texture, sieved soils (<2 mm) were treated repeatedly with 10 ml of hydrogen peroxide solution (30%) to remove organic matter until no bubbles were produced. The residues were boiled with 10 ml of hydrochloric acid (HCl; 10%) to remove lime, rinsed repeatedly with deionized water to pH 7 and then dispersed in 10 ml of sodium hexametaphosphate (0.5 M) by sonication (30 s). Particle size distribution was determined using Malvern Mastersizer 2000 particle analyzer^62^. Clay is defined as particles smaller than 2 µm.

### Analyses of amino sugars and lignin phenols

Amino sugars were analyzed following the classic recipe of Zhang and Amelung^63^. Briefly, ~0.5 g of air-dried soil (<2 mm) was hydrolyzed with 10 mL of 6 M HCl at 105 °C for 8 h. The cooled sample was added with 100 μg of myo-inositol as a surrogate standard, filtered, evaporated to dryness at 50–53 °C under reduced pressure and re-dissolved in 5 mL of MilliQ water with the pH adjusted to 6.6–6.8 using 1 M potassium hydroxide. Precipitates were discarded after centrifugation (1000×*g*, 10 min) and the supernatant was freeze-dried. Amino sugars were re-dissolved in methanol and separated from salts by centrifugation. After the addition of a quantitative standard (methyl-glucamine), amino sugars were transformed into aldononitrile derivatives by heating in 0.3 ml of a derivatization reagent containing 32 mg ml^−1^ of hydroxylamine hydrochloride and 40 mg ml^−1^ of 4-(dimethylamino)pyridine in pyridine and methanol mixture (4:1; v-v) at 75 °C for 30 min^14,64^. The derivatives were further acetylated with 1 ml of acetic anhydride at 75–80 °C for 20 min and mixed with 1.5 ml of dichloromethane after cooling. Excessive derivatization reagents were removed by extracting with HCl (1 M) and MilliQ water while the dichloromethane phase containing amino sugar derivatives were dried under nitrogen before quantification.

For lignin phenol analysis, approximately 2–5 g of dried soil or 0.1 g plant tissue was first solvent-extracted and base hydrolyzed to remove extractable and hydrolysable lipids, respectively^65^. Dried residues were then subjected to copper (II) oxide (CuO) oxidation. Briefly, soil (2 g) or plant residues (50 mg) were mixed with 1 g CuO, 100 mg ammonium iron (II) sulfate hexahydrate [Fe(NH_4_)_2_(SO_4_)_2_·6H_2_O] and 20 mL of nitrogen-purged NaOH solution (2 M) in teflon-lined bombs. All bombs were flushed with nitrogen in the headsapce for 10 min and heated at 150 °C for 2.5 h. The lignin oxidation products (LOPs) were spiked with a surrogate standard (ethyl vanillin), acidified to pH 1 with 6 M HCl, and kept in the dark for 1 h. After centrifugation (2500 r.p.m., 30 min), LOPs were liquid–liquid extracted from the clear supernatant with ethyl acetate, and concentrated under nitrogen. LOPs were derivatized with N,O-bis-(trimethylsilyl) trifluoroacetamide (BSTFA) and pyridine (70 °C, 1 h) to yield trimethylsilyl (TMS) derivatives before quantification.

Biomarkers of interest were quantified using internal standards on a Trace 1310 gas chromatograph coupled to an ISQ mass spectrometer (Thermo Fisher Scientific, USA) using a DB-5MS column (30 m × 0.25 mm i.d., film thickness, 0.25 μm). For amino sugars, the oven temperature was held at 120 °C for 1 min, increased to 250 °C at a rate of 10 °C min^−1^ for 2.5 min with final isothermal hold at 270 °C for 5.5 min. For lignin phenols, the temperature was held at 65 °C for 2 min, increased from 65 to 300 °C at a rate of 6 °C min^−1^ with final isothermal hold at 300 °C for 20 min. Helium was used as carrier gas (0.8 mL min^−1^). The mass spectrometer was operated in the electron impact mode (EI) at 70 eV and scanned from 50 to 650 Da. Quantification was achieved by comparing with surrogate standards to account for compound loss during extraction procedures. External quantification standards were used to normalize the response factor for different amino sugars and lignin phenols separately.

Amino sugars were summarized as glucosamine, galactosamine, mannosamine, and muramic acid. Among them, glucosamine is more abundant in fungal than bacterial cell walls while muramic acid is considered to trace bacteria-derived carbon^39,40^. The other two amino sugars are ubiquitous. Vanillyl (vanillin, acetovanillone, vanillic acid), syringyl (syringaldehyde, acetosyringone, syringic acid), and cinnamyl (*p*-coumaric acid, ferulic acid) phenols were summarized to represent lignin in the soil. Biomarker concentration was normalized to SOC content to reflect its relative abundance in SOC.

### Klason lignin analysis for plant tissues

Klason lignin was analyzed for the aboveground and belowground biomass samples (~3 g) using the standard procedure^30^. Briefly, dried vegetation samples were hydrolyzed with 72% sulfuric acid in pressurized tubes in a water bath at 30 ± 3 °C for 1 h with occasional stirring. The sulfuric acid was then diluted to 4% in the tubes and the sealed samples were autoclaved for 1 h at 121 °C. After cooling, the hydrolysates were removed and all remaining solids were transferred into a filtering crucible and rinsed with >50 mL of deionized water. After drying to a constant weight at 105 ± 3 °C, the precipitated solids were determined gravimetrically as Klason lignin.

### Literature data

For biomarker concentrations in other grasslands, we used the Web of Science to search for papers containing words such as ‘grassland’ and ‘amino sugar’ or ‘lignin phenol’ published in 1990–2016. We used data from 24 papers employing similar extraction protocols (i.e., the method of Zhang and Amelung^63^ for amino sugars and CuO oxidation in Teflon bombs at 170 ± 2 °C, 2–2.5 h for lignin phenols). Soil properties (including soil pH, SOC, N, and texture) associated with the corresponding sites were also collected. To examine clay’s influence on amino sugars, references using the same method for clay measurement (i.e., sieving and centrifugation) were included^14,20,24,25^. Climatic (including aridity index) data was obtained from the aforementioned databases accordingly (Supplementary Data 2).

### Data and statistical analysis

Differences in biomarker concentrations, ratios and soil properties were examined by one-way ANOVA among varied vegetation types and *t* test between Mongolian and non-Mongolian grasslands. Relationships of biomarker concentrations or ratios with environmental parameters were tested by Pearson correlation using mean values of three site replicates after natural-log transformation of non-normally distributed data. If normal distribution was not achieved after data transformation, two-tailed Spearman linear correlation was used instead. Differences or correlations were considered to be significant at a level of *p* < 0.05.

SEM was fitted by maximum likelihood estimation using the ‘lavaan’ package^66^ of R software (version 3.3.2). Given the nested sampling design, sites were included as a block effect to account for site-level effects using the ‘lavaan.survey’ package^67^. For the category of environmental variables encompassing more than one parameter, a principal component analysis (PCA) was conducted and its feasibility was checked using Kaise-Meyer-Olkin (KMO) test and Bartlett test of sphericity (BS). The test result (Supplementary Table 5) indicates that PCA is appropriate to use for our data^68^. Hence, the first principal component was used to represent the corresponding category of variables (explaining >75% of variations in all cases; Supplementary Table 6).

The a prior model was evaluated and optimized by step-wise exclusion of variables with non-significant regression weights and step-wise inclusion of additional correlations based on the modification indices and goodness of fit for the initial model^69^. Due to our relatively small dataset with non-normal distributions, the models were modified with Satorra–Bentler correction to improve the chi-square approximation of goodness-of-fit test statistics^70^ and confirmed using the Bollen–Stine bootstrap test. Models are considered to have a good fit when the bootstrap *P* value is within 0.1–1.0^71^. We checked the bivariate relationships between all variables to ensure that a linear model was appropriate (Supplementary Fig. 10). Correlations were included in the SEM (Supplementary Fig. 11) but not shown in Fig. 5 for clarity. It should be mentioned that the ratio of samples to model parameters was low for our SEMs (especially for lignin phenols). Hence, the SEM results were further confirmed by other statistical methods.

Partial correlation analysis was conducted to further support the importance of aridity with other environmental variables being controlled, and vice versa. Multiple stepwise regression analysis was also employed to identify the most important variable(s) affecting the distribution of biomarkers and (Ad/Al)_S_ ratios in the Mongolian soils. Only variables showing significant correlations with the investigated parameter were included as independent variables in the model and were excluded in the subsequent selection process based on the *p* values (i.e., only variables with *p* ≤ 0.10 were retained in the model). After the establishment of regression models, normal distribution of model residues was checked while collinearity among the selected variables was avoided based on the variance inflation factor (a cutoff value of 1 was chosen when collinearity was considered not to exist) to ensure robustness of the models.

### Data availability

The data that support the findings of this study are available in the Supplementary Information.

## Electronic supplementary material

Supplementary Information

Description of Additional Supplementary Files

Supplementary Data 1

Supplementary Data 2

Supplementary Data 3
