# Supplementary material for: Divergent accumulation of microbial necromass and plant lignin components in grassland soils
Source: Nat Commun. 2018 Aug 28;9:3480. doi: 10.1038/s41467-018-05891-1 (PMC6113315; doi:10.1038/s41467-018-05891-1)
Supplement: Supplementary file 1 — Supplementary Information [file 41467_2018_5891_MOESM1_ESM.pdf]

Supplementary Information

Divergent Accumulation of Microbial Necromass and Plant Lignin Components in Grassland Soils

Ma *et al.*

Supplementary Table 1: Standardized partial regression coefficient of the multiple stepwise regression analysis for amino sugar and lignin phenol concentrations with soil, climatic and vegetation variables in the Mongolian grasslands. Mean values of three site replicates are used.

**Variable**	**Amino sugars (n =38)**	**Lignin phenols (n = 13)**
Aridity index	0.48	−0.75
Aboveground biomass	ns	ns
Belowground biomass	ns	ns
Soil organic carbon	ns	ns
Soil nitrogen	ns	ns
pH	ns	ns
Phospholipid fatty acids	ns	ns
Clay	ns	ns
Iron (Fe)	ns	ns
Aluminum (Al)	ns	ns
**R^2 of model**	**0.23**	**0.57**
p	**< 0.01**	**< 0.01**

Supplementary Table 2: Values of r for the partial correlation analysis between environmental variables and amino sugar concentration ($p < 0.05$). Mean values of three site replicates are used.

Tested variable	Controlled variable							
	Aridity index	AGB	BGB	SOC	N	Clay	pH	Microbial PLFAs
Aridity index	na	0.30*	0.46	ns	ns	0.51	0.41	0.40
AGB	ns	na	0.34	ns	ns	0.44	ns	ns
BGB	ns	ns	na	ns	ns	0.30	ns	ns
SOC	ns	ns	0.44	na	ns	0.35	0.41	0.43
N	ns	ns	0.40	ns	na	0.50	0.37	0.37
Clay	ns	ns	ns	ns	ns	na	ns	ns
pH	ns	ns	ns	ns	ns	−0.34	na	ns
Microbial PLFAs	ns	ns	ns	ns	ns	−0.35	ns	na

AGB, aboveground biomass; BGB, belowground biomass; SOC, soil organic carbon; N, nitrogen; PLFAs, phospholipid fatty acids; Fe, iron; Al, aluminum ; na, not applicable; ns, not significant. *Marginally significant ($p = 0.07$).

Supplementary Table 3: Values of r for the partial correlation analysis between environmental variables and lignin phenol concentration ($p < 0.05$). Mean values of three site replicates are used.

Tested variable	Controlled variable								
	Aridity index	AGB	BGB	SOC	N	Fe	Al	pH	Microbial PLFAs
Aridity index	na	−0.75	−0.62	−0.63	−0.65	−0.72	−0.70	−0.61	−0.73
AGB	ns	na	ns	ns	ns	ns	−0.58	ns	ns
BGB	ns	ns	na	ns	ns	−0.68	−0.72	ns	−0.58
SOC	ns	ns	ns	na	ns	−0.70	−0.70	ns	ns
N	ns	ns	ns	ns	na	−0.66	−0.67	ns	ns
Fe	ns	ns	ns	ns	ns	na	ns	ns	ns
Al	ns	ns	ns	ns	ns	ns	na	ns	ns
pH	ns	ns	ns	ns	ns	0.82	0.81	na	0.59
Microbial PLFAs	ns	ns	ns	ns	ns	0.68	0.63	ns	na

AGB, aboveground biomass; BGB, belowground biomass; SOC, soil organic carbon; N, nitrogen; PLFAs, phospholipid fatty acids; Fe, iron; Al, aluminum ; na, not applicable; ns, not significant.

Supplementary Table 4. Standardized partial regression coefficients of the multiple stepwise regression analysis for the acid-to-aldehyde (Ad/Al) ratio of syringyl (S) phenols with environmental variables in the Mongolian grasslands (n = 13). Mean values of three site replicates are used.

Variable	$(Ad/Al)_S$
**Aridity index**	0.71
**Aboveground biomass**	ns
**Soil organic carbon**	ns
**Nitrogen**	ns
**R^2 of model**	**0.51**
p	**< 0.01**

Supplementary Table 5. Results of Kaise-Meyer-Olkin (KMO) test and Bartlett test of sphericity (BS) for variables used for principle component analysis (PCA) in the paper.

Dataset	Parameters	KMO value	BS test
Amino sugars (Mongolia)	SOC, N	0.50	$\chi^2 = 352$; $p < 0.05$
	AGB, BGB	0.50	$\chi^2 = 41$; $p < 0.05$
Lignin phenols (Mongolia)	SOC, N	0.50	$\chi^2 = 62$; $p < 0.05$
	AGB, BGB	0.50	$\chi^2 = 13$; $p < 0.05$
	Fe, Al	0.50	$\chi^2 = 35$; $p < 0.05$

SOC, soil organic carbon; N, nitrogen; AGB, aboveground biomass; BGB, belowground biomass; Fe, iron; Al, aluminum.

Supplementary Table 6. Correlations (r values) of individual parameters to the first principal component of different variable groups used in the structural equation modelling (SEM) of biomarkers in Mongolian grassland soils based on principal component analysis ($p < 0.05$).

**Variables**	**Amino sugars**	**Lignin phenols**
**Variable group: Soil C & N**		
SOC (mg g^{-1})	0.995	0.98
N (mg g^{-1})	0.995	0.98
Variance explained	***99%***	***95%***
**Variable group: Plant**		
AGB (kg m^{-2})	0.88	0.88
BGB (kg m^{-2})	0.88	0.88
Variance explained	***78%***	***77%***
**Variable group: Soil mineral**		
Fe (%)	na	0.95
Al (%)	na	0.95
Variance explained	***na***	***89%***

SOC, soil organic carbon; N, nitrogen; AGB, aboveground biomass; BGB, belowground biomass; Fe, iron; Al, aluminum; na, not applicable.

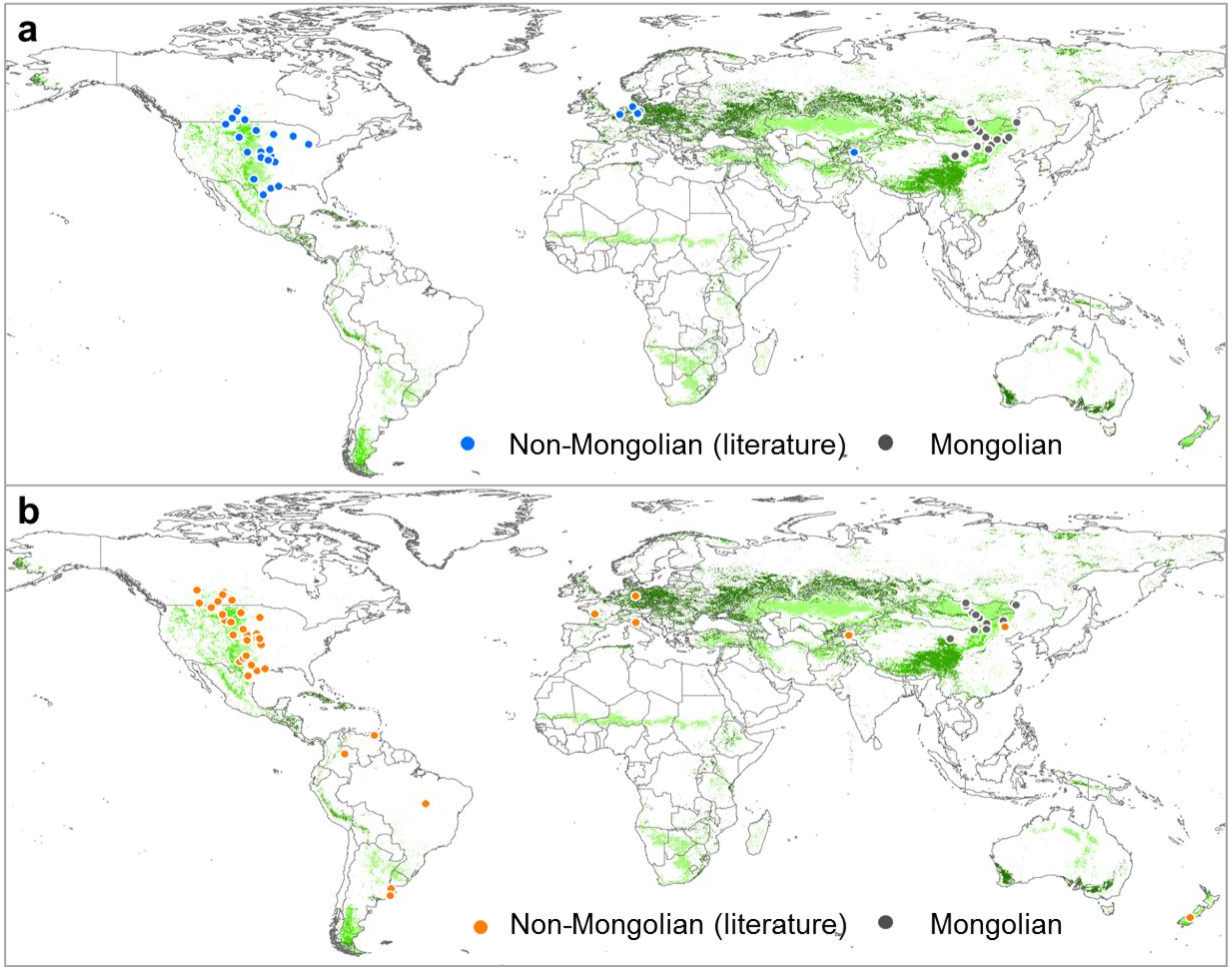

Supplementary Figure 1. Site distribution for all amino sugar (a) and lignin phenol data (b) used in this paper. Detailed list of references for the literature data is in Supplementary Table 2. Land cover classification map of grasslands is derived from the Global Land Cover Characteristics Database v2.0 (https://lta.cr.usgs.gov/GLCC).

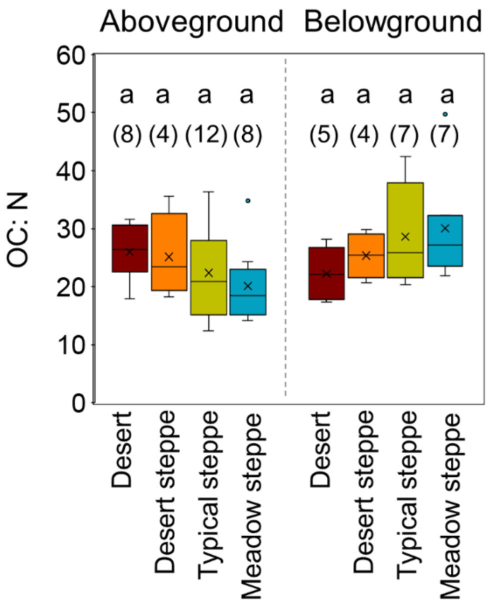

Supplementary Figure 2. Ratios of organic carbon-to-nitrogen (OC:N) in the overlaying vegetation of Mongolian soils. Solid line and cross in the box mark the median and mean of each dataset, respectively. The upper and lower ends of boxes denote the 0.25 and 0.75 percentiles, respectively. The upper and lower whisker caps denote the maximum and minimum values, respectively. Dots denote outliners. Numbers in parenthesis indicate the number of samples. Lower-case letter *a* indicates the same level for all samples ($p > 0.05$).

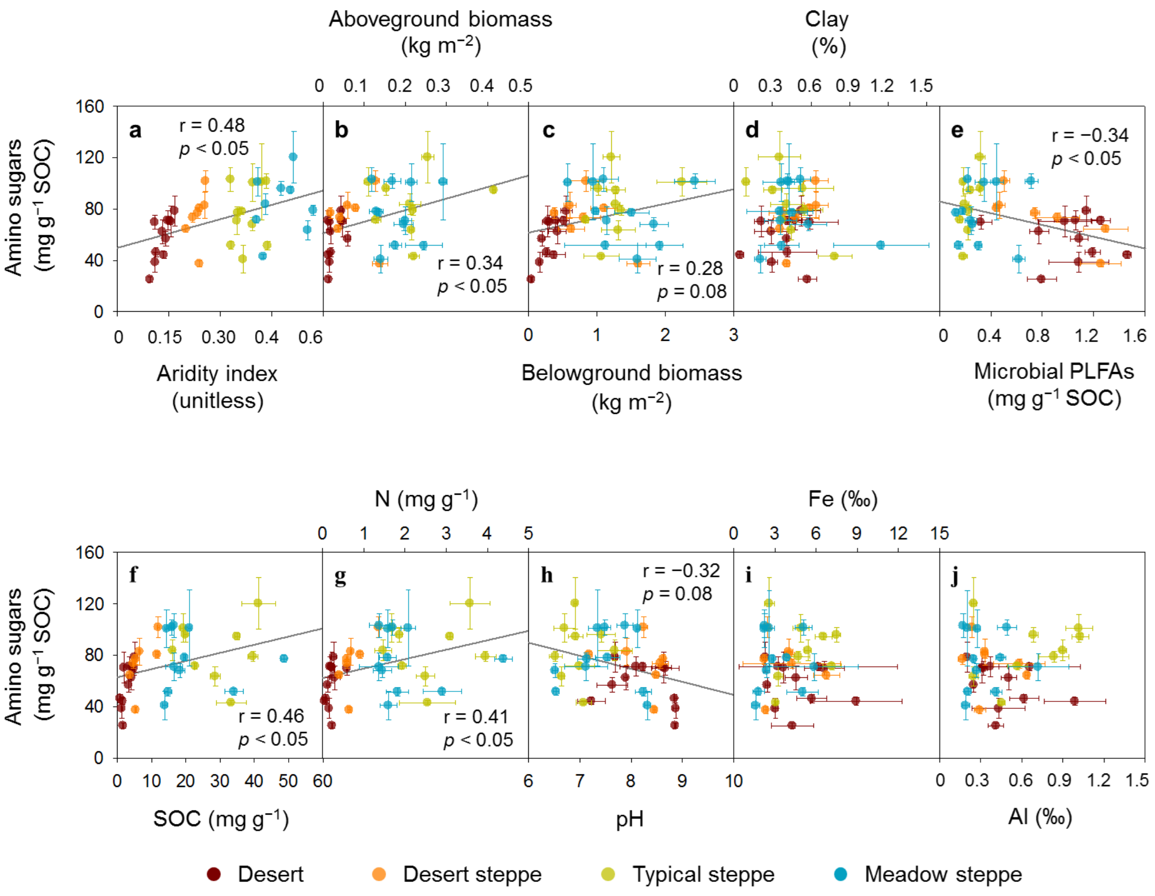

Supplementary Figure 3. Spearman correlations between the soil organic carbon (SOC)-normalized concentrations of amino sugars and environmental variables in the Mongolian grasslands (n = 38). Error bars represent standard error of mean for three site replicates. PLFAs, phospholipid fatty acids; N, nitrogen; Fe, iron; Al, aluminum.

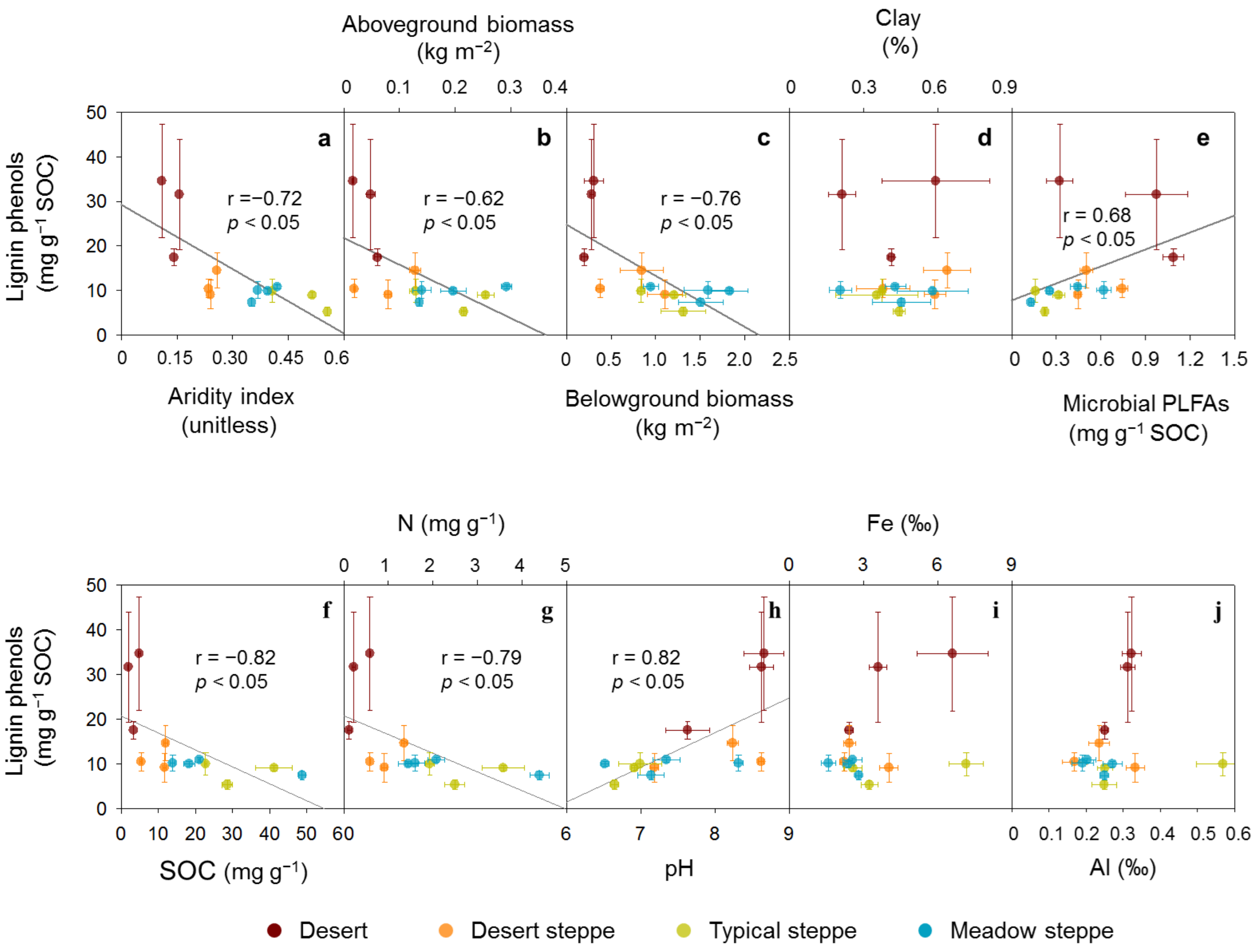

Supplementary Figure 4. Spearman correlations between the soil organic carbon (SOC)-normalized concentrations of lignin phenols and environmental variables in the Mongolian grasslands (n = 13). Error bars represent standard error of mean for three site replicates. PLFAs, phospholipid fatty acids; N, nitrogen; Fe, iron; Al, aluminum.

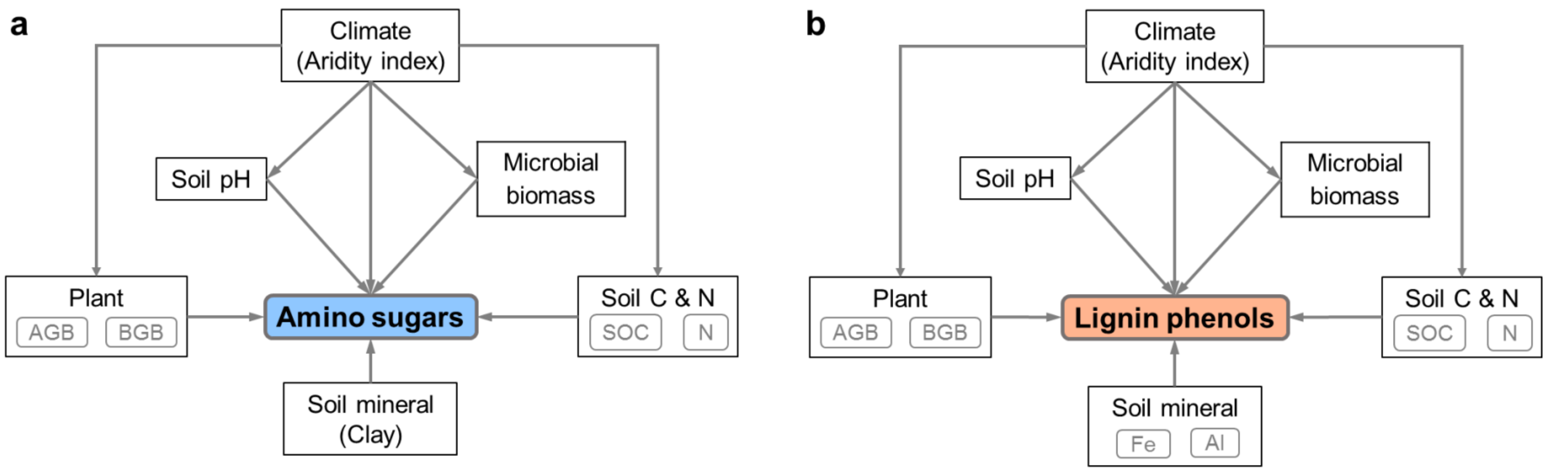

Supplementary Figure 5. A priori models for the structural equation modeling analysis of soil organic carbon (SOC)-normalized concentrations of amino sugars (a) and lignin phenols (b) in the Mongolian grasslands**.** Arrows indicate flows of causality based on knowledge. Environmental variables are categorized into plant, soil carbon (C) & nitrogen (N), and soil mineral by the principle component analysis. AGB, aboveground biomass; BGB, belowground biomass; Fe, iron; Al, aluminum; PLFAs, phospholipid fatty acids. Please note that correlations between variables embedded in the original data are not shown here.

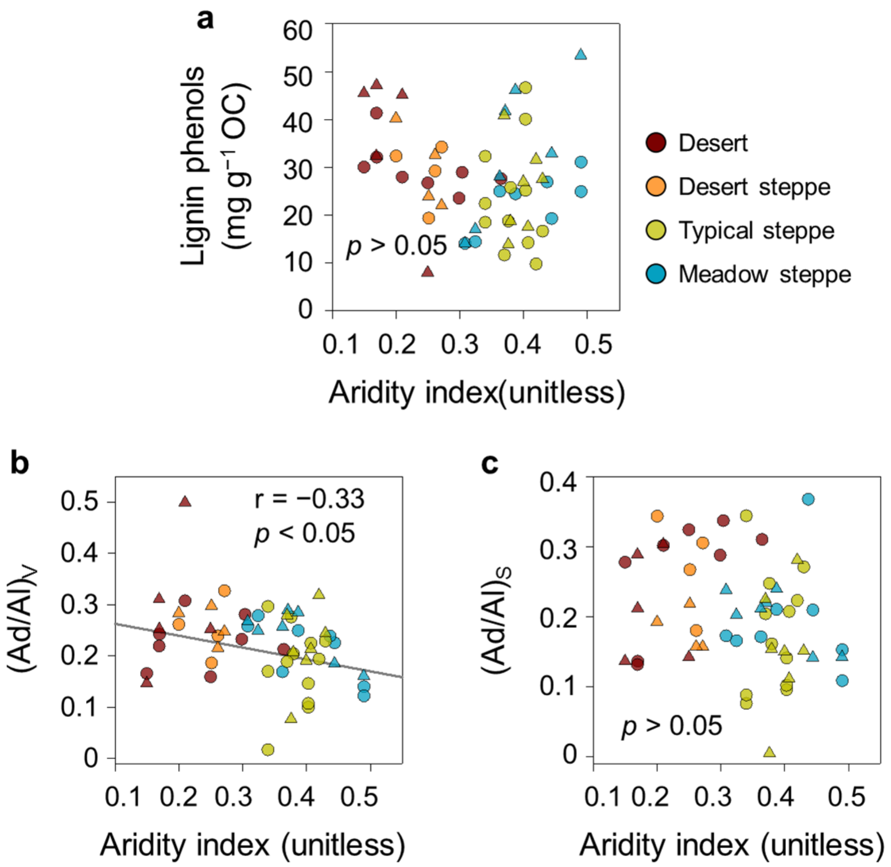

Supplementary Figure 6. Variations in lignin phenol concentrations (a) and the acid-to-aldehyde (Ad/Al) ratios of vanillyl (V; b) and syringyl (S; c) phenols in plant tissues with aridity index in the Mongolian grasslands. Dots and triangles represent the aboveground (n = 32) and belowground biomass (n = 23) samples, respectively. Black line represents Spearman correlation ($p < 0.05$). OC, organic carbon.

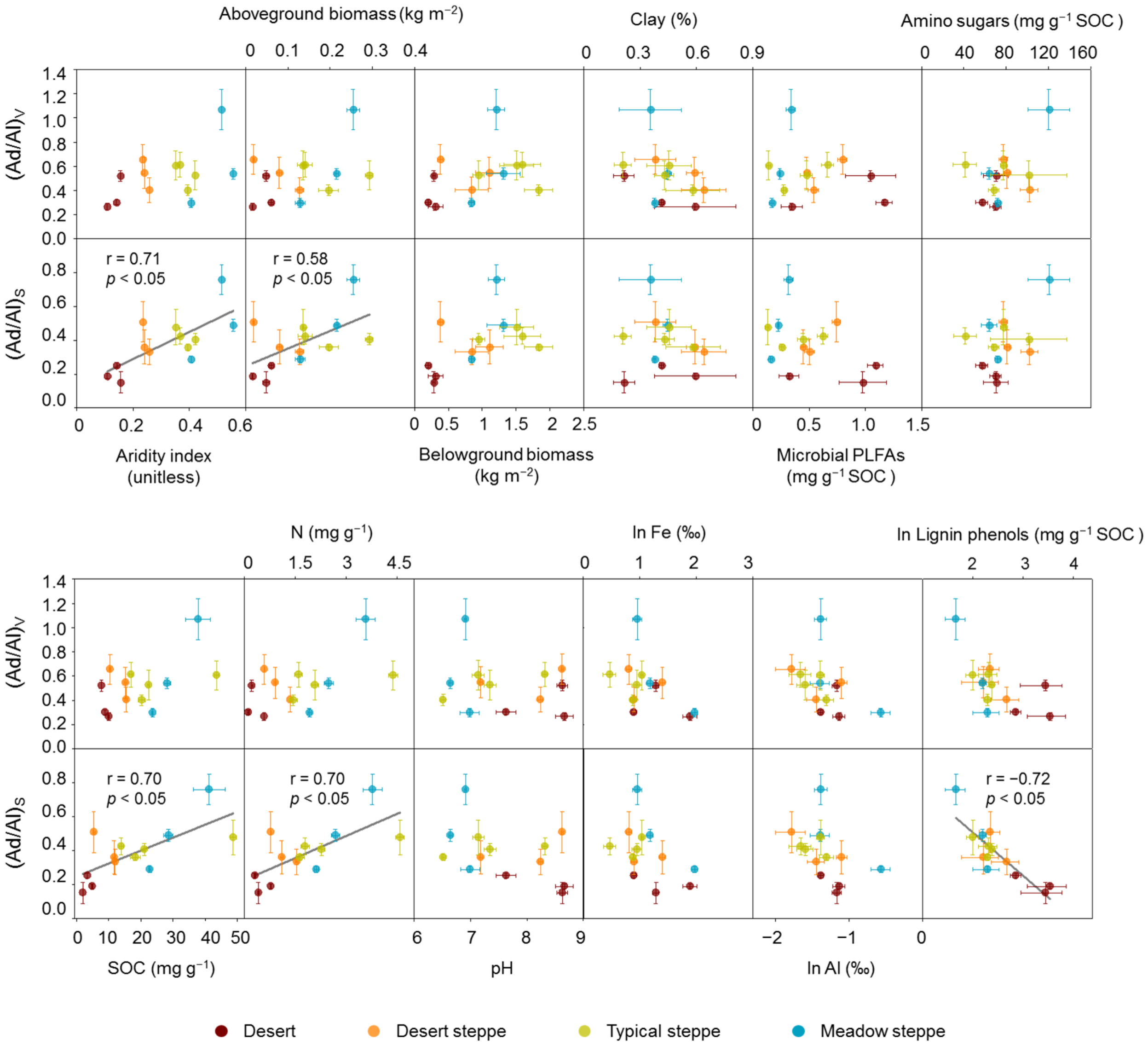

Supplementary Figure 7. Pearson correlations of the acid-to-aldehyde (Ad/Al) ratios of both vanillyl (V) and syringyl (S) phenols with different variables along the Mongolian grasslands (n = 39). PLFAs, phospholipid fatty acids; SOC, soil organic carbon; N, nitrogen; Fe, iron; Al, aluminum.

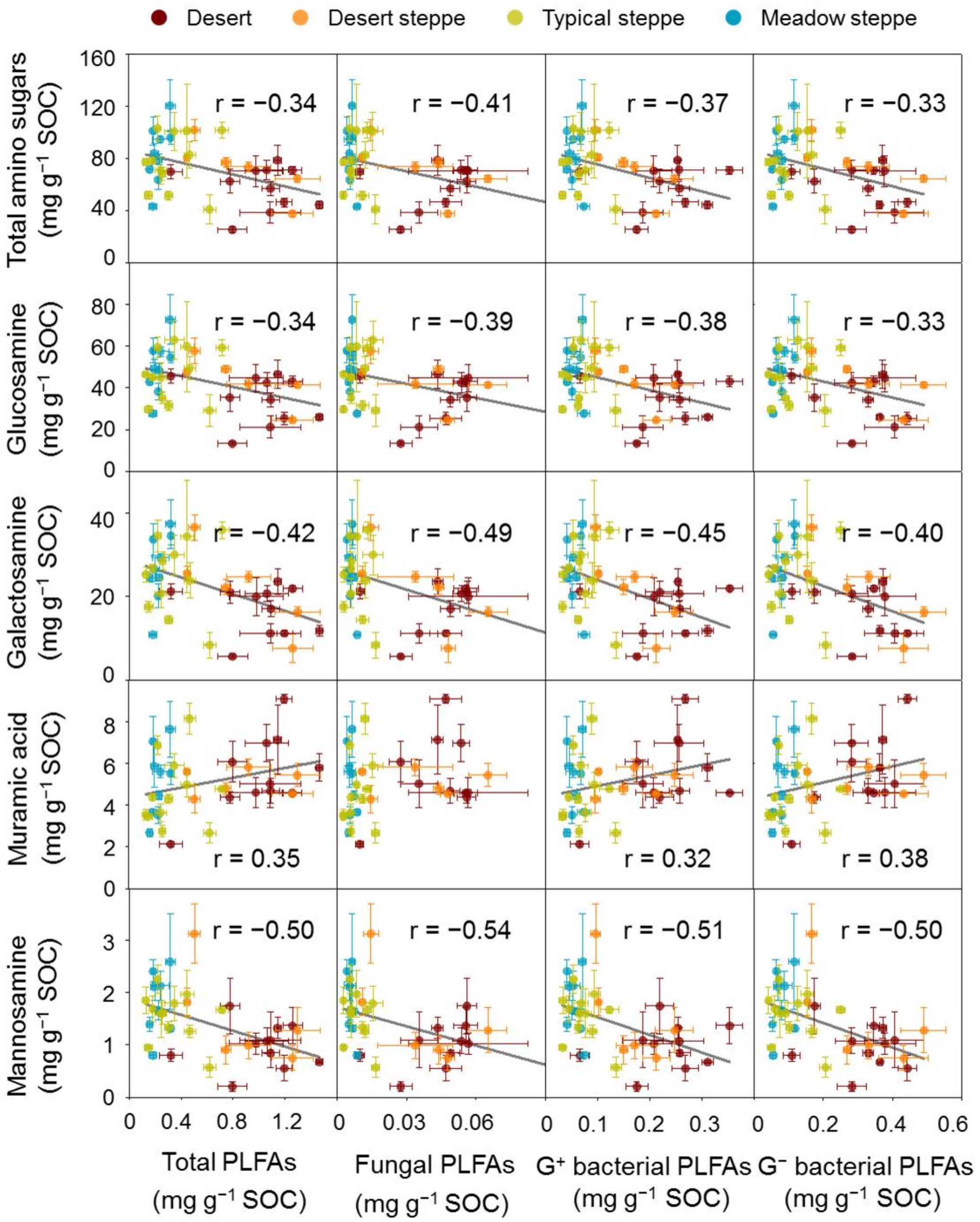

Supplementary Figure 8. Spearman correlations of total and individual amino sugars with total and subgroups of phospholipid fatty acids (PLFAs) in the Mongolian soils (n = 113; $p < 0.05$). SOC, soil organic carbon; G^+, gram-positive; G^-, gram-negative.

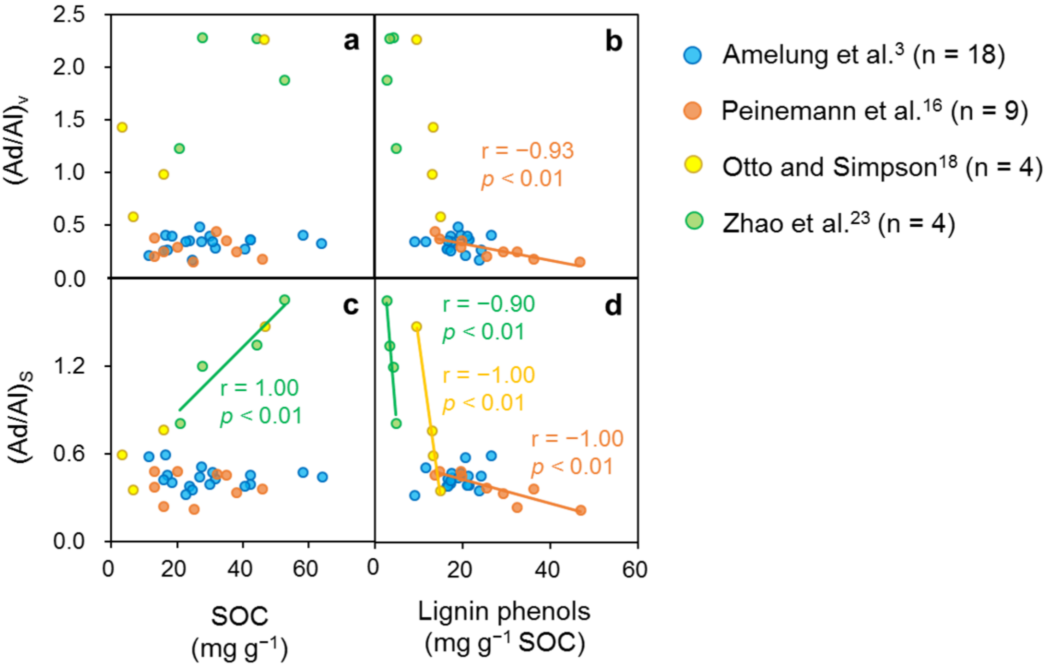

Supplementary Figure 9: Spearman's correlation of lignin acid-to-aldehyde (Ad/Al) ratios for vanillyl (V; a-b) and syringyl (S; c-d) phenols with soil organic carbon (SOC) and lignin phenol concentrations in the published data for other grassland soils. Colored line represents linear correlation for the corresponding dataset.

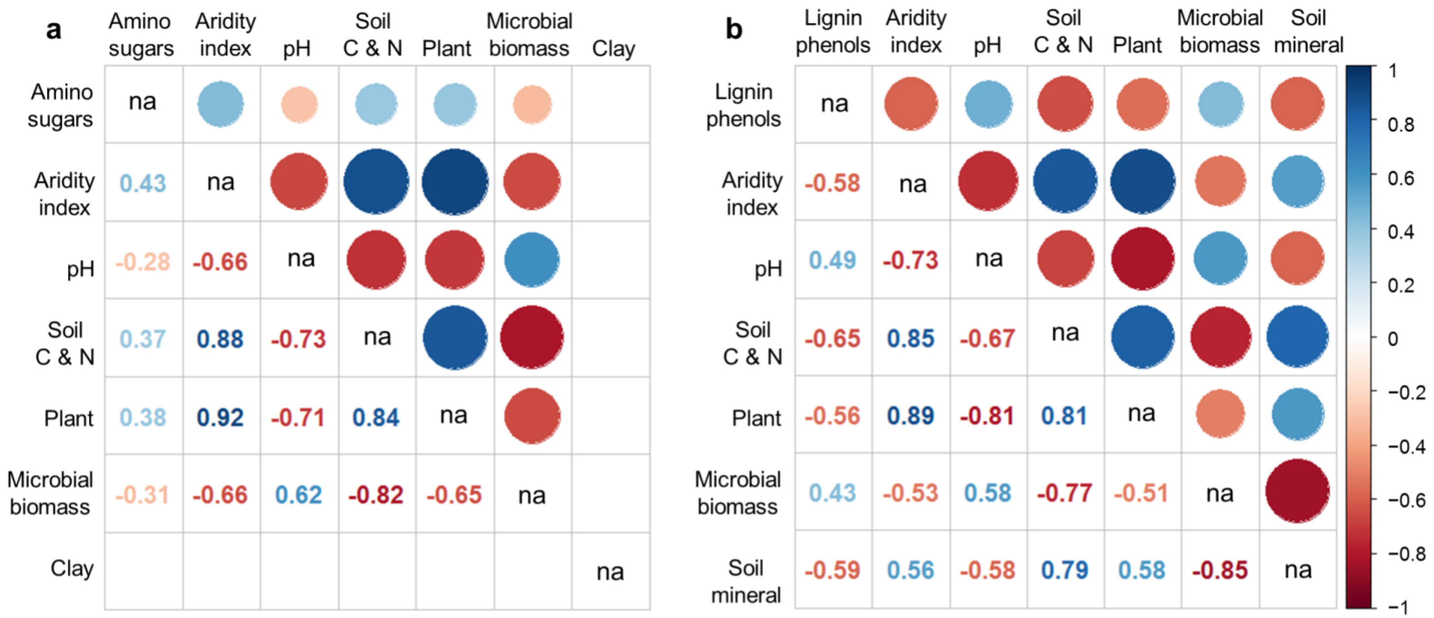

Supplementary Figure 10: Correlation matrix of environmental variables used in the SEM for amino sugars (a; n = 113) and lignin phenols (b; n = 39). Numbers in the lower left triangle indicate Spearman correlation coefficients (r) between the corresponding variables ($p < 0.05$), proportional to the size of the colored dots in the upper right triangle. Empty cells indicate non-significant correlations ($p > 0.05$). SOC: soil organic carbon; N: nitrogen; PLFA: phospholipid fatty acid; Fe: iron; Al: aluminum; na: not applicable. Soil C & N, plant and soil mineral are defined by a principle component analysis (Supplementary Table 9).

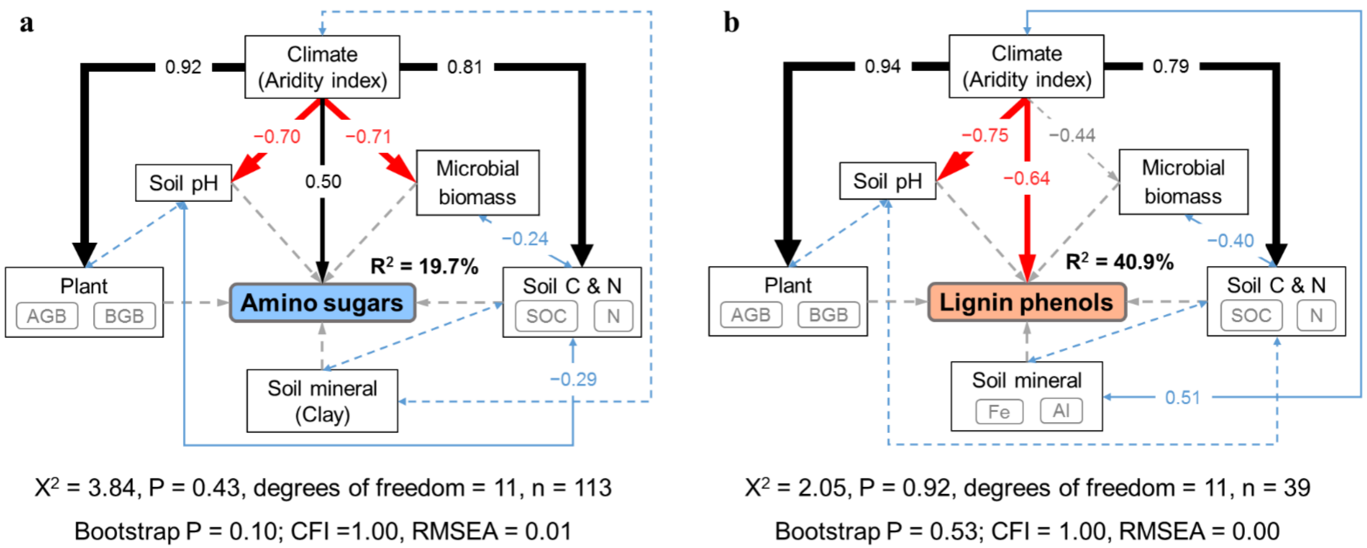

Supplementary Figure 11: Best-supported structural equation models showing all correlations and causal pathways between variables for the soil organic carbon (SOC)-normalized concentrations of amino sugars (a) and lignin phenols (b) in the Mongolian grasslands. Black and red arrows indicate positive and negative flows of causality ($p < 0.05$), respectively. Grey dotted lines indicate insignificant pathways from a priori models (Fig. S5). Blue arrows and blue dotted lines indicate significant ($p < 0.05$) and insignificant correlations ($p > 0.05$) between variables, respectively. Numbers on the single-headed arrows indicate significant standardized path coefficients, proportional to the arrow width. Numbers on the double-headed arrows are partial correlation coefficients. All other parameters are defined in Figure 5 in the main text.

SUPPLEMENTARY REFERENCES

1. Zhang, X. D. & Amelung, W. Gas chromatographic determination of muramic acid, glucosamine, mannosamine, and galactosamine in soils. *Soil Biol. Biochem.* **28,** 1201–1206 (1996).
2. Amelung, W., Zhang, X. D., Flach, K. W. & Zech, W. Amino sugars in native grassland soils along a climosequence in North America. *Soil Sci. Soc. Am. J.* **63,** 86–92 (1999).
3. Amelung, W., Flach, K. W. & Zech, W. Lignin in particle–size fractions of native grassland soils as influenced by climate. *Soil Sci. Soc. Am. J.* **63,** 1222–1228 (1999).
4. Zhang, X. D. *et al.* Land–use effects on amino sugars in particle size fractions of an Argiudoll. *Appl. Soil Ecol.* **11,** 271–275 (1999).
5. Glaser, B., Turrión, M. & Alef, K. Amino sugars and muramic acid—biomarkers for soil microbial community structure analysis. *Soil Biol. Biochem.* **36,** 399–407 (2004).
6. Decock, C. *et al.* Critical assessment of the applicability of gas chromatography–combustion–isotope ratio mass spectrometry to determine amino sugar dynamics in soil. *Rapid Commun. Mass Spectrom.* **23,** 1201–1211 (2009).
7. Bode, S., Denef, K. & Boeckx, P. Development and evaluation of a high–performance liquid chromatography/isotope ratio mass spectrometry methodology for delta13C analyses of amino sugars in soil. *Rapid Commun. Mass Spectrom.* **23,** 2519–2526 (2009).
8. Liang, C. *et al.* Soil microbial residue storage linked to soil legacy under biofuel cropping systems in southern Wisconsin, USA. *Soil Biol. Biochem.* **57,** 939–942 (2013).
9. Liang, C. *et al.* Switchgrass rhizospheres stimulate microbial biomass but deplete microbial necromass in agricultural soils of the upper Midwest, USA. *Soil Biol. Biochem.* **94,** 173–180 (2016).
10. Khan, K. S. *et al.* Microbial biomass, fungal and bacterial residues, and their relationships to the soil organic matter C/N/P/S ratios. *Geoderma* **271,** 115–123 (2016).
11. Guggenberger, G., Christensen, B. T. & Zech, W. Land–use effects on the composition of organic matter in particle–size separates of soil: I. Lignin and carbohydrate signature. *Eur. J. Soil Sci.* **45,** 449–458 (1994).
12. Guggenberger, G., Zech, W. & Thomas, R. J. Lignin and carbohydrate alteration in

particle–size separates of an oxisol under tropical pastures following native savanna. *Soil Biol. Biochem.* **27,** 1629–1638 (1995).

13. Amelung, W., Zech, W. & Flach, K. W. Climatic effects on soil organic matter composition in the great plains. *Soil Sci. Soc. Am. J.* **61,** 115–123 (1997)
14. Glaser, B. *et al.* Soil organic matter quantity and quality in mountain soils of the Alay Range, Kyrgyzia, affected by land use change. *Biol. Fert. Soils* **31,** 407–413 (2000).
15. Knicker, H. *et al.* Soil organic matter transformations induced by Hieracium pilosella L. in tussock grassland of New Zealand. *Biol. Fert. Soils* **32,** 194–201 (2000).
16. Peinemann, N., Guggenberger, G. & Zech, W. Soil organic matter and its lignin component in surface horizons of salt–affected soils of the Argentinian Pampa. *Catena* **60,** 113–128 (2005).
17. Grünewald, G., Kaiser, K., Jahn, R. & Guggenberger, G. Organic matter stabilization in young calcareous soils as revealed by density fractionation and analysis of lignin–derived constituents. *Org. Geochem.* **37,** 1573–1589 (2006).
18. Otto, A. & Simpson, M. J. Evaluation of CuO oxidation parameters for determining the source and stage of lignin degradation in soil. *Biogeochemistry* **80,** 121-142 (2006)
19. Rumpel, C. & Chabbi, A. Response of bulk chemical composition, lignin and carbohydrate signature to grassland conversion in a ley–arable cropping system. *Nutr. Cycl. Agroecosys.* **88,** 173–182 (2009).
20. Clemente, J. S., Simpson, A. J. & Simpson, M. J. Association of specific organic matter compounds in size fractions of soils under different environmental controls. *Org. Geochem.* **42,** 1169–1180 (2011).
21. Rückamp, D., Martius, C., Bragança, M. A. L. & Amelung, W. Lignin patterns in soil and termite nests of the Brazilian Cerrado. *Appl. Soil Ecol.* **48,** 45–52 (2011).
22. Pisani, O., Hills, K. M., Courtier–Murias, D., Simpson, A. J., et al. Molecular level analysis of long term vegetative shifts and relationships to soil organic matter composition. *Org. Geochem.* **62,** 7–16 (2013).
23. Zhao, L., Wu, W., Xu, X. & Xu, Y. Soil organic matter dynamics under different land use in grasslands in Inner Mongolia (northern China). *Biogeosciences* **11,** 5103–5113 (2014).

24. Pisani, O. *et al.* Molecular composition of soil organic matter with land–use change along a bi–continental mean annual temperature gradient. *Sci. Total Environ.* **573,** 470–480 (2016).
