# Supplementary material for: Divergent accumulation of microbial necromass and plant lignin components in grassland soils
Source: Nat Commun. 2018 Aug 28;9:3480. doi: 10.1038/s41467-018-05891-1 (PMC6113315; doi:10.1038/s41467-018-05891-1)
Supplement: Supplementary file 2 — Description of Additional Supplementary Files [file 41467_2018_5891_MOESM2_ESM.pdf]

Description of Additional Supplementary Files

Supplementary Data 1: Information on sampling sites and raw data in the Mongolian grasslands (mean ± s.e.m.; Supplementary Data file uploaded separately).

Supplementary Data 2: Published data of amino sugar and lignin phenol concentrations in the surface soils (0–10 cm) of grasslands over the world (Supplementary Data file uploaded separately).

Supplementary Data 3: Sample information and lignin composition of plant biomass from varied vegetation types along the Mongolian transects (Supplementary Data file uploaded separately).
